# Classical monocytes maintain ex vivo glycolytic metabolism and early but not later inflammatory responses in older adults

**DOI:** 10.1186/s12979-019-0143-1

**Published:** 2019-01-26

**Authors:** Brandt D. Pence, Johnathan R. Yarbro

**Affiliations:** 1 School of Health Studies, Memphis, TN 38152 USA; 2 Center for Nutraceutical and Dietary Supplement Research, University of Memphis, Memphis, TN 38152 USA; 3 304 Elma Roane Fieldhouse, University of Memphis, Memphis, TN 38152 USA

**Keywords:** Immunosenescence, Inflammaging, Immunometabolism, Glycolysis, Metabolism, Innate immunity, Inflammation

## Abstract

**Background:**

Inflammaging is a condition of chronic low-grade inflammation due to the aging process and is associated with a variety of chronic diseases. Monocytes are innate immune cells which contribute to inflammation and are dysregulated during aging, demonstrated reduced phagocytosis, increased inflammation, and alterations in subset proportions. Metabolism is known to determine immune cell function, with quiescent and anti-inflammatory cells primarily relying on fatty acid oxidation, while activated and inflammatory cells primarily rely on glycolysis. We have previously shown an age-related decrease in mitochondrial respiratory capacity in monocytes, so we hypothesized here that a compensatory shift toward glycolysis would occur which would also exacerbate inflammation.

**Results:**

Using Seahorse assays, we profiled glycolysis in classical monocytes isolated from older (60–80 yr) and younger (18–35 yr) adults. Aging did not affect parameters of basal glycolysis in the glycolysis stress test, nor did it alter glycolytic activation early (2 h) or later (24 h) post-LPS stimulation. Cytokine gene expression was unchanged between aged and young subjects at 2 h post-LPS but was reduced in older subjects at 24 h post-LPS either significantly (*IL1B*) or near-significantly (*IL6*, *IL10*).

**Conclusions:**

Aging appears not to affect glycolytic metabolism ex vivo in classical monocytes, but may reduce cytokine expression at later timepoints. Studies examining monocytes stimulated with age-altered circulating factors or with other pattern recognition receptor agonists may shed further light on monocyte metabolism as a determinant of immunosenescence and inflammaging.

**Electronic supplementary material:**

The online version of this article (10.1186/s12979-019-0143-1) contains supplementary material, which is available to authorized users.

## Introduction

The adult population of the United States is aging at an increasing rate, with nearly 20% of U.S. adults expected to be over age 65 by 2030 [1]. Physiological aging is associated with a variety of chronic diseases with inflammatory etiology, to the extent that the term ‘inflammaging’ has been coined to describe the link between aging, inflammation, and disease [2]. Investigators in biogerontology have ascribed this condition to a number of possible causes, including sterile inflammation due to cellular damage [2], innate immune activation due to adaptive immunosenescence [3], mitochondrial dysfunction [4], and alterations in the gut microbiome [5] among others. Many of these changes have been associated with inflammaging experimentally, but the condition is likely to be multifactorial and to vary depending on individual. Therefore, experiments which seek to delineate the mechanisms underlying inflammaging are still of great importance, especially if they lead to therapeutic strategies designed to ameliorate age- and inflammation-related disease.

A key player in age-related innate immune dysfunction is the monocyte, a circulating cell with important functions including antigen presentation, phagocytosis, and cytokine production [6]. Dysregulation of monocyte phenotype and/or function is associated with chronic diseases including but not limited to various cancers [7] and cardiovascular and renal diseases [8, 9]. Aging alters monocyte subpopulation proportions and integrin expression patterns and increases basal cytokine production [10, 11]. Despite this, the mechanisms underlying age-related monocyte dysfunction are not well-understood.

Immunometabolic processes have become important avenues of inquiry in the last decade. It is now clear that the majority of immune cell types upregulate glycolytic ATP production during activation in order to meet enhanced energy demands [12]. While this has received increased attention in recent years, monocytes have long been known to utilize glycolysis during cellular activation by lipopolysaccharide, with studies dating back at least to the mid-1980s having documented the phenomenon [13–15]. To our knowledge, despite studies demonstrating altered basal and LPS-stimulated cytokine responses in monocytes from older adults [11], the impact of aging on parameters of glycolysis in monocytes has not been investigated. However, we recently demonstrated mitochondrial dysfunction in monocytes isolated from older adults [16], which we speculate may reduce the ability of these cells to utilize oxidative energy production and therefore may dispose them to a greater reliance on glycolysis. Therefore, we hypothesized that monocytes from older adults would show greater levels of basal and activated glycolysis, and that this would be associated with greater inflammation.

## Methods

### Subjects

We recruited young (18–35 yr) and older (60–80 yr) subjects from the Greater Memphis area. Subjects were not excluded on the basis of race, sex, or socioeconomic status, but were excluded if they had a previous diagnosis of inflammatory or metabolic diseases. Medication use is described in Additional file 1: Table S1. Subjects were recruited in two cohorts as the 2 h LPS assay described below was added after initial data collection was complete. Cohort 1 subjects are the same individuals as those reported in our previous paper [16]. Not all subjects from cohort 1 were available for follow-up testing, so for cohort 2 several new subjects were recruited to replace cohort 1 subjects who were no longer available. Overall, *N* = 3 young and *N* = 6 aged individuals participated in both cohorts. All protocols were approved by the Institutional Review Board at the University of Memphis (protocol #4361).

### Monocyte isolation

Monocytes were isolated as previously described [16] from 8 to 16 ml EDTA-treated whole blood using an immunomagnetic negative selection kit (StemCell Technologies, Vancouver, CAN). Subjects fasted overnight prior to each blood collection. Our previous work [16] demonstrated that aging had no impact on monocyte purity resulting from the isolation process, with both groups averaging around 90% purity by CD14 staining and flow cytometry. The isolation kit includes CD16 depletion, thus only classical monocytes are isolated, which was confirmed by flow cytometry in our previous publication [16]. We conducted all downstream assays or lysed cells immediately after cell isolation; no cells were frozen for later analysis.

### Extracellular flux assays

We conducted several analyses for glycolysis, based on extracellular acidification rate (ECAR), on a Seahorse XFp analyzer (Agilent, Santa Clara, CA). For all assays, 1.5 × 10^5^ monocytes were plated per well in duplicate or triplicate on a sterile XFp plate coated with 0.56 μg Cell-Tak (Corning Inc., Corning, NY) per well. All plates were incubated at 37 °C in a non-CO_2_ incubator for 1 h prior to analysis on the XFp system to stabilize plate CO_2_ levels. For all assays, ECAR was expressed relative to cell number, as determined by microscopy with manual counting at 10× using an inverted microscope, in order to correct for technical variance in cell seeding density. All cell counts were obtained immediately following the Seahorse assay and immediately preceding the processing for gene expression analysis (if applicable).

**Fig. 1:**
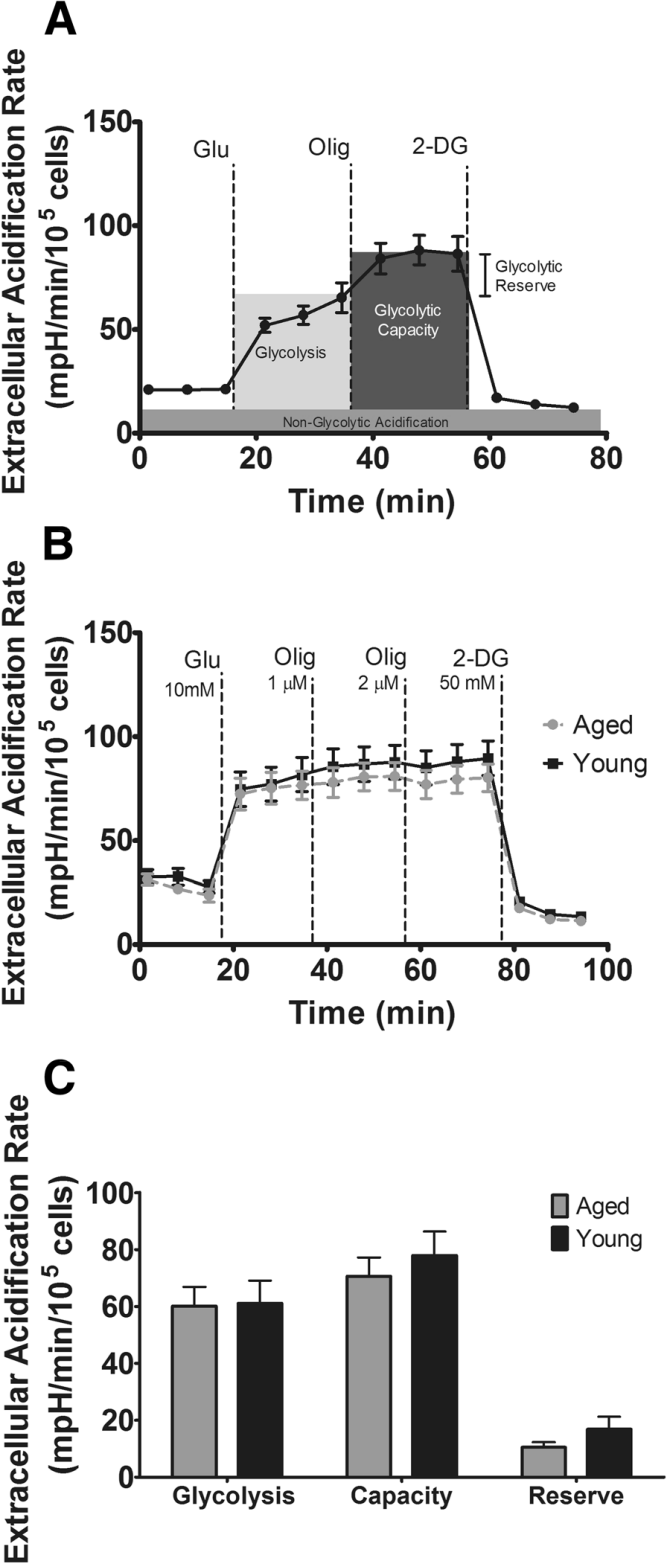
Glycolysis stress test. **a** Example glycolysis stress test assay showing calculated parameters. On the Y-axis, extracellular acidification rate is the mpH change over 1-min intervals, which is then expressed relative to 100,000 monocytes to account for between-well cell number variation. **b** Aged vs. young glycolysis stress test. **c** Calculated parameters of glycolysis stress test for aged and young subjects

The Glycolysis Stress Test was performed based on manufacturer’s instructions with serial stimulations in the following sequence: (A) 10 mM glucose; (B) 1 μM oligomycin; (C) 1 μM oligomycin (2 μM final concentration); (D) 50 mM 2-deoxyglucose. The non-standard dual oligomycin injection was performed on advice from technical support to overcome a delayed monocyte response to oligomycin seen during optimization. Parameters calculated from the Glycolysis Stress Test include basal glycolysis, glycolytic capacity, glycolytic reserve, and non-glycolytic acidification. A visualization of parameter calculations is shown in Fig. 1a. The assay strategy was adapted from previous work in monocytes [17].

To determine the immediate glycolytic response to an inflammatory stimulus, we stimulated XFp-plated monocytes with 10 ng·ml^− 1^ lipopolysaccharide (LPS) and analyzed ECAR over the course of 2 h. This protocol was adapted from a previous report by Raulien et al. [18]. The difference between minimal pre- and maximal post-LPS ECAR was compared between aged and young to determine whether aging affected the magnitude of the response. To determine differences in the kinetic response, ECAR area under the curve (AUC) was calculated by the trapezoidal method. Following the assay, supernatants were removed, and cells were lysed with 100 μl Trizol (Thermo Fisher Scientific, Waltham, MA) per well and stored at -80 °C for later analysis of LPS-stimulated cytokine gene expression.

To determine if aging affected glycolysis in the longer term, we stimulated XFp-plated monocytes for 23 h with 1 ng·ml^− 1^ LPS. Following a subsequent 1 h CO_2_ stabilization (i.e. at 24 h post-LPS), we analyzed ECAR on the XFp system, then removed supernatants, lysed cells with 100 μl Trizol per well, and stored lysates at -80 °C until analysis for cytokine gene expression.

### Cytokine expression

Cell lysates in Trizol from the 2 h and 24 h LPS incubation Seahorse assays were processed for RNA isolation using the Trizol method based on manufacturer’s instructions. Isolated RNA was quantified by nanospectrophotometry on a Nanodrop Lite instrument (Thermo Fisher Scientific). For each sample, 0.5 μg RNA was reverse-transcribed to cDNA using a commercially-available kit (High-Capacity cDNA Reverse Transcription Kit, Thermo Fisher Scientific). Gene expression was determined between groups by quantitative real-time polymerase chain reaction (qPCR) on a QuantStudio 6 instrument (Thermo Fisher Scientific). For each sample, 25 ng (2 h LPS assay) or 4 ng (24 h LPS assay) of cDNA was assayed in duplicate using a TaqMan master mix (TaqMan Fast Advanced Master Mix, Thermo Fisher Scientific). Pre-validated primer/probe sets were purchased from Thermo Fisher Scientific. The specific gene expression assays were as follows: *B2M* (Hs00187842_m1), *IL1B* (Hs01555410_m1), *TNF* (Hs00174128_m1), *IL12A* (Hs01073447_m1), *IL6* (Hs00174131_m1), *IL10* (Hs00961622_m1). *IL12A* gene expression was low (average Ct > 30) and is not reported.

### Data analysis

All data were analyzed in R v. 3.3.3 (R Foundation for Statistical Computing, Vienna, AUT). Categorical demographic data (race, sex) were analyzed by Chi-square test. Continuous demographic and anthropometric data (age, height, weight, body mass index) were analyzed by independent-samples T test between young and older subjects. Glycolysis stress test parameters, the magnitude and AUC kinetic responses for the 2 h LPS stimulation, and cytokine gene expression for the 2 h LPS assay were analyzed by independent-samples T test between young and older subjects. Longer-term 24 h LPS responses were measured by two-way factorial analysis of variance in a 2 × 2 (stimulation condition × group) design. Data analyzed by independent samples T test had equal variance by Levene’s test. A significance level of *p* < 0.05 was used for all tests. Reported results are mean ± SEM.

### Protocol availability

Step-by-step protocols for Seahorse-based assays [19–21] and for monocyte isolation [22] are available at protocols.io.

## Results

### Data availability

The datasets and analytical scripts supporting the conclusions of this article are available in the FigShare repository [23].

### Subject characteristics

Subject demographic and anthropometric data are shown in Table 1. In both cohorts of subjects, groups differed significantly on age as expected, but did not differ significantly on other demographic or anthropometric measures.

**Table 1:** Demographic and Anthropometric Characteristics of Subjects

*Cohort 1*			
	Aged (*N* = 9)	Young (*N* = 9)	Prob
Age, yr. (range)	65.0 ± 1.2 (61–71)	25.7 ± 1.9 (18–33)	*p* < 0.001
Height, cm (range)	176.7 ± 3.0 (164–192)	169.2 ± 4.1 (157–189)	*p* = 0.166
Weight, kg (range)	79.6 ± 3.6 (57–93)	77.3 ± 8.6 (48–129)	*p* = 0.814
BMI, kg/m^2^ (range)	25.5 ± 1.1 (20–32)	26.4 ± 1.9 (19–36)	*p* = 0.688
Female, N (%)	4 (44)	5 (56)	*p* = 0.637
White, N (%)	5 (56)	6 (67)	
Black, N (%)	4 (44)	2 (22)	Race: *p* = 0.415
Hispanic, N (%)	0 (0)	1 (11)	
*Cohort 2*			
	Aged (N = 9)	Young (*N* = 11)	Prob
Age, yr. (range)	66.6 ± 1.4 (60–72)	27.5 ± 1.3 (20–34)	*p* < 0.001
Height, cm (range)	173.8 ± 3.0 (163.0–191.0)	169.8 ± 3.2 (155.0–191.0)	*p* = 0.375
Weight, kg (range)	77.3 ± 4.0 (54.4–99.8)	74.8 ± 7.5 (43.4–133.8)	*p* = 0.789
BMI, kg/m^2^ (range)	25.6 ± 1.2 (18.8–31.6)	25.7 ± 2.0 (17.4–36.7)	*p* = 0.980
Female, N (%)	4 (44)	4 (36)	*p* = 0.714
White, N (%)	7 (78)	4 (36)	
Black, N (%)	2 (22)	2 (18)	Race: *p* = 0.059
Asian, N (%)	0 (0)	5 (45)	

Cohort 1: glycolysis stress test and 24 h LPS assays. Cohort 2: 2 h LPS assay. Yr, year; cm, centimeters; kg, kilograms; BMI, body mass index; N, number of subjects

### Extracellular flux assays and cytokine expression

To determine the effect of aging on parameters of glycolysis absent stimulation, we conducted a Seahorse Glycolysis Stress Test (Fig. 1a). Aging had no effect on responses to the serial injections (Fig. 1b) and did not alter calculated parameters (Fig. 1c) of glycolysis (*p* = 0.927), glycolytic capacity (*p* = 0.508), or glycolytic reserve (*p* = 0.228).

**Fig. 2:**
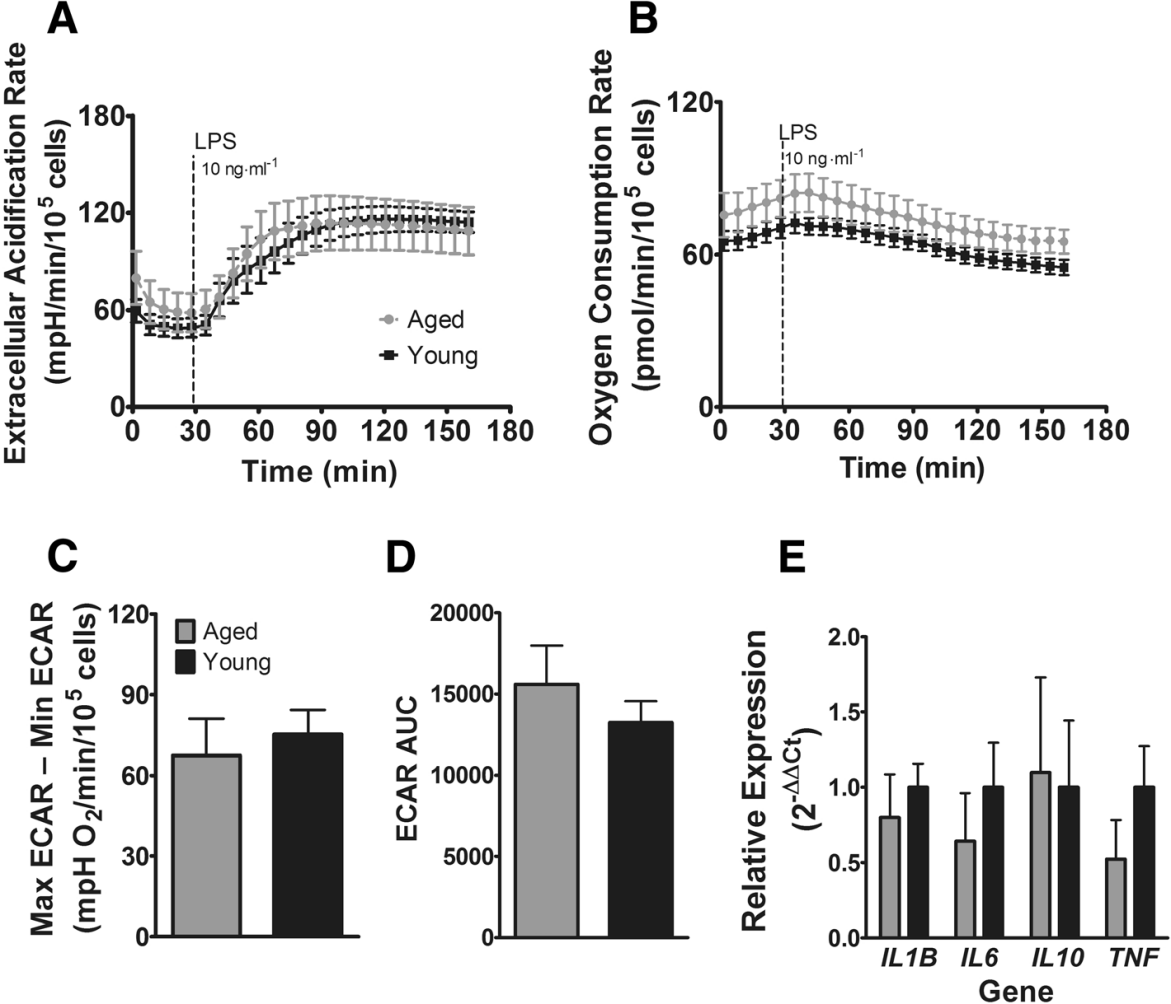
Early metabolic and inflammatory responses to LPS. **a** Glycolytic response to LPS in aged and young subjects. **b** Oxygen consumption response to LPS in aged and young subjects. **c** Magnitude of glycolytic response (maximum ECAR - minimum ECAR). **d** Kinetic glycolysis response (AUC). **e** Cytokine gene expression. ECAR: extracellular acidification rate. AUC: area under the curve

To determine the effect of aging on the immediate response to LPS, we stimulated monocytes for 2 h with 10 ng·ml^− 1^ LPS and analyzed ECAR and oxygen consumption rate (OCR) (Fig. 2a & b). Aging did not affect the magnitude of the ECAR response as calculated by the difference between maximal ECAR after LPS and ECAR at baseline (*p* = 0.640, Fig. 2c). Aging additionally did not affect ECAR kinetics as measured by AUC (*p* = 0.401, Fig. 2d) and did not affect the magnitude of the gene expression response to LPS (Fig. 2e) for *IL1B* (*p* = 0.520), *IL6* (*p* = 0.365), *IL10* (*p* = 0.624), or *TNF* (*p* = 0.180). Although the OCR response was slightly higher across all time points in the aged group, this was not significant, and both groups showed the characteristic decrease in oxygen consumption after LPS stimulation (Fig. 2b).

**Fig. 3:**
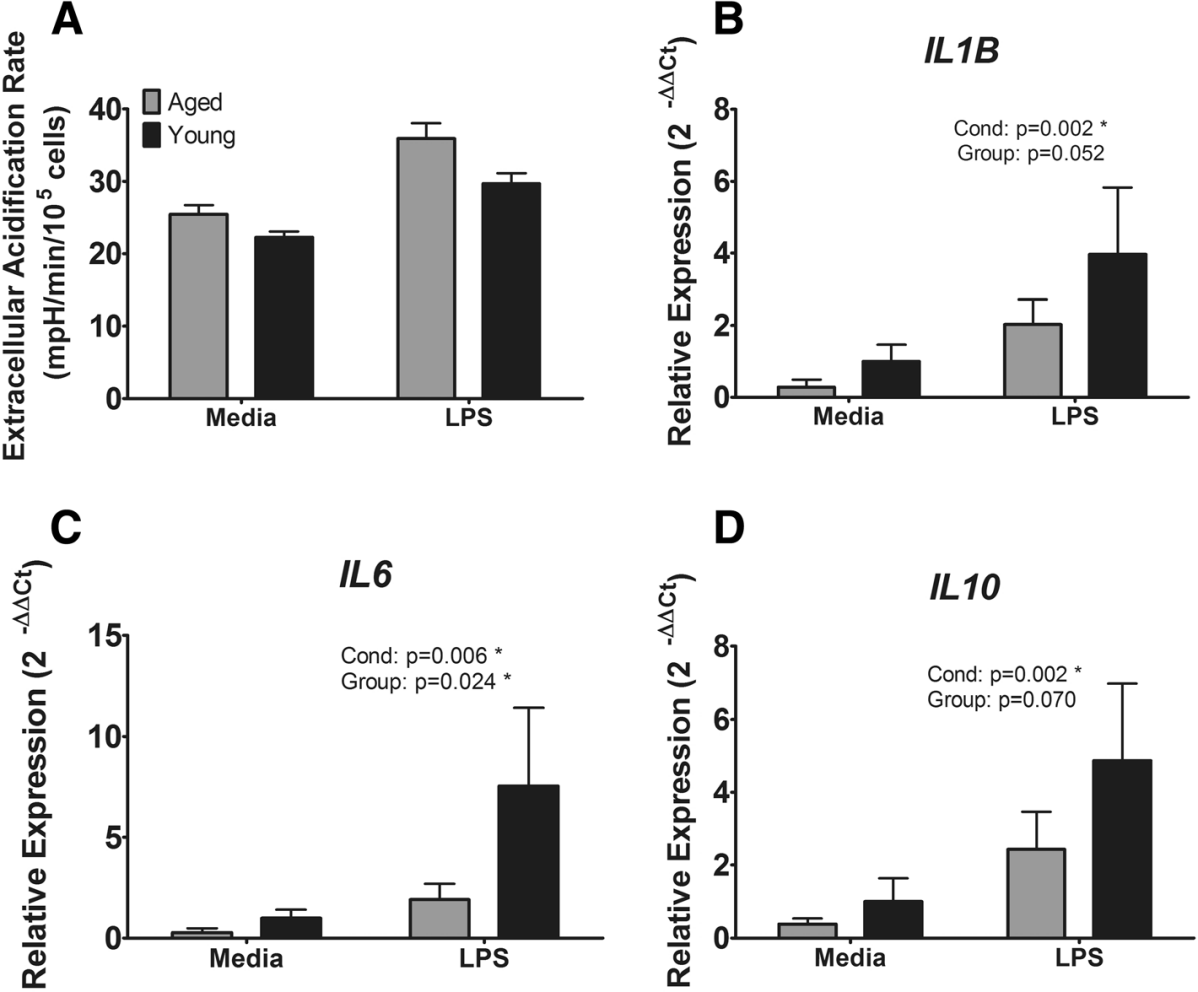
Later metabolic and inflammatory responses to LPS. **a** Glycolytic response to LPS at 24 h post-stimulation. **b** Gene expression of *IL1B*. **c** Gene expression of *IL6*. **d** Gene expression of *IL10*. Cond: LPS vs. Media main effect. Group: Aged vs. Young main effect. * *p* < 0.05

To examine whether aging affects the duration of glycolytic activation, we stimulated monocytes with LPS for 23 h and measured ECAR following a 1 h non-CO_2_ equilibration (Fig. 3a). LPS non-significantly increased ECAR (*p* = 0.096) compared to medium. There was no effect of age (*p* = 0.401) and no age × condition interaction (*p* = 0.797). Inflammatory gene expression at 24 h post-stimulation was increased in the LPS condition for *IL1B* (*p* = 0.002, Fig. 3b), *IL6* (*p* = 0.006, Fig. 3c), and *IL10* (p = 0.002, Fig. 3d) and trended toward a decrease in *TNF* (*p* = 0.067, not shown). Additionally, there was a significant main effect of age for *IL6* (*p* = 0.024) and near-significant main effects for *IL1B* (*p* = 0.052) and *IL10* (*p* = 0.070) such that aged individuals had reduced expression of these genes. No significant age × condition interactions were found for any measured genes.

## Discussion

We have previously demonstrated that aging impairs mitochondrial respiratory capacity in classical monocytes [16]. Immune cells require increased ATP production to meet energy demands during activation or inflammation, for the purposes of cytokine production, chemotaxis, proliferation, and a host of other processes [12]. We hypothesized that this would increase the cellular demand for glycolysis and therefore drive increased inflammation, but in this study we demonstrated no aging effects on ex vivo basal glycolytic parameters or on ex vivo glycolytic activation by LPS either early (2 h) or later (24 h) after stimulation. Additionally, aging did not alter the early inflammatory response to LPS, but we did find a significant reduction in gene expression of interleukin (IL)-6 and near-significant reductions in gene expression of IL-1β and IL-10 in aged monocytes at 24 h post-LPS. Although immunometabolism is well-established as a field, its application to the field of aging is relatively new. The effect of aging on increasing oxidative stress and reactive oxygen species generation is well-established as a pillar of aging [24] with obvious metabolic underpinnings, and the effect of altering metabolism through dietary means has been a recent area of interest in immunogerontology [25], but studies examining the direct aging effect on substrate metabolism in stimulated or unstimulated immune cells are still uncommon. We have reported reductions in mitochondrial respiratory capacity in classical monocytes [16], and naïve T cells from aged mice have been shown to have similar deficiencies [26]. Because many age-related changes such as epigenetic modifications and alterations in sirtuin activity have well-known metabolic linkages, and because metabolic alterations are known to effect immune cell phenotypic changes characteristic of the aging process, studies in this area are likely to be very important to extending our understanding of inflammaging and immunosenescence in the immediate future.

Previous studies examining cytokine production in monocytes during aging have had mixed results. Hearps et al. demonstrated that intracellular tumor necrosis factor (TNF)-α protein expression was increased after LPS treatment in aged subjects [11], and Mariani et al. showed similar upregulation of CCL3 and CCL5 due to LPS stimulation of monocytes in nonagenarians [27]. However, cytokine gene expression due to LPS stimulation of isolated monocytes from older adults has also been demonstrated to be reduced [28, 29], unchanged [30], or differentially-expressed based on cytokine measured [31] in several additional studies. These disparate findings may result in part from monocyte isolation strategy, in addition to other potential sources of variation such as subject number, population demographics, age ranges, etc.

Of note, our purification technique includes CD16 depletion and isolates only the classical monocyte subpopulation [16], while many other studies use density-gradient centrifugation or other techniques which isolate all monocyte subpopulations. LPS reactivity varies by monocyte subset [32, 33], and we and others have shown age-related alterations in monocyte subset frequency in humans [11, 16, 34, 35]. Because of this age-related increase in CD16+ monocyte proportions, our reported findings do not fully encapsulate the potential age-related effects on monocyte glycolytic responses. However, because purification strategy may bias LPS response measures, we argue that consideration of monocyte subpopulations in isolation is the most meaningful method to detecting relevant age-related changes in function and is an important characteristic to consider when designing or evaluating studies on aging and human monocytes. Ultimately, the variable effects of LPS stimulation on aged monocyte responses in previous studies may be explained by purification strategies which yield all 3 subsets of monocytes, thereby biasing responses one way or another depending on subset frequency. To our knowledge our study is unique in that it is the first to study the effect of aging on LPS responses in a purified subset of monocytes, which is a considerable strength of our design but does not obviate the need for further study into the effect of aging on monocyte function in the intermediate and non-classical subsets.

Our study was limited to inflammatory stimulation with the toll-like receptor (TLR)-4 agonist LPS. Activation of monocytes by agonists targeting other pattern recognition receptors may show different effects. For example, aging has been observed to reduce TNFα and IL-6 expression by approximately half when monocytes are stimulated with the TLR1/2 agonist Pam3CSK4 [36]. Recently, Pam3CSK4 was shown to induce different metabolic effects than LPS, with Pam3CSK4 upregulating and LPS downregulating oxidative phosphorylation [37]. As such, although we demonstrated no aging effect on metabolism due to LPS stimulation in this study, investigations using different inflammatory stimuli may yield different results.

Several subjects in both the aged and young groups reported use of medications which may influence metabolic outcomes (Additional file 1: Table S1). In cohort 2, two aged subjects reported statin use, while a single aged subject who participated in both cohorts reported using metformin and thyroid medication, albeit without a formal diabetes diagnosis. Several subjects in both the aged and young groups reported use of allergy medication, which have immunomodulatory properties. These medications may have altered our metabolic and inflammatory outcome measures, which is a limitation. We are underpowered to statistically detect differences due to medication use in this study, although the medications listed here appeared to have little effect on our study parameters upon cursory analysis. Older individuals who are not on at least one medication are not particularly common, and we suggest that our subject pool is representative of the typical aged population. Regardless, this represents a limitation that is not easily addressable in human aging research, as aging studies in either healthy or typical populations are mutually-exclusive but both beneficial. Confirmatory studies in rodent models may also partially address this limitation.

Because in our previous work we showed differences in mitochondrial respiration only under maximizing conditions (i.e. FCCP stimulation), we suggest that we did not detect differences in glycolysis because aged monocytes do not demonstrate significant impairments in metabolism during ex vivo stimulation, except when a severe stressor such as FCCP is applied. Our study is limited to ex vivo measures of metabolic and inflammatory functions, as well as to gene expression differences as the sole measure of inflammation. Although this is a common strategy, it necessarily isolates monocytes from their natural environment. Interestingly, although we found no changes in glycolysis at either immediate or later time points post-LPS, gene expression of several cytokines was reduced at the later time point. In many immune cells including monocytes and macrophages, glycolytic activation is critical to pro-inflammatory responses [12], so this was a somewhat unexpected finding. It is likely that downstream age-related effects such as epigenetic regulation of gene expression may be responsible for these seemingly glycolysis-independent effects, as aging is well-established to alter epigenetic signatures in immune cells [38].

A recent comprehensive review of the effects of aging on monocytes and macrophages noted that the in vivo environment in aging may be more relevant to cellular function [10]. For example, aged individuals have increased circulating IL-6 levels, and genetic ablation of IL-6 in aged mice resulted in a stronger inflammatory response in macrophages [39]. However, given the difference in monocyte biology between mice and humans, this bears further study. A recent comprehensive proteomics study revealed large age-associated changes in a number of circulating factors, including several known to modulate myeloid cell function [40]. Therefore, study of monocytes in their native environment, either in vivo or in culture using conditioned media, is likely to yield further insights into the effect of aging on monocyte function. Additionally, other outcomes related to monocyte function and inflammation, including reactive oxygen species production, phagocytosis, or chemotaxis, may be modulated by age-related changes in cellular metabolism and require further investigation.

## Conclusions

In summary, we demonstrated no effect of aging on ex vivo glycolytic metabolism of monocytes either under basal or LPS-stimulated conditions. Additionally, cytokine gene expression was unchanged at early timepoints post-LPS but was significantly or near-significantly decreased for IL-1β, IL-6, and IL-10 at a later (24 h) timepoint. Future research examining metabolic changes due to alternate pattern recognition receptor agonists is likely to yield additional insights, as are studies which better replicate in vivo conditions.

## Additional file

Additional file 1: **Table S1.** Medication Use. (DOCX 12 kb)

## Abbreviations

AUC: area under the curve
ECAR: extracellular acidification rate
IL: interleukin
LPS: lipopolysaccharide
TLR: toll-like receptor
TNF: tumor necrosis factor
