# Supplementary material for: Classical monocytes maintain ex vivo glycolytic metabolism and early but not later inflammatory responses in older adults
Source: Immun Ageing. 2019 Jan 26;16:3. doi: 10.1186/s12979-019-0143-1 (PMC6348080; doi:10.1186/s12979-019-0143-1)
Supplement: Supplementary file 1 — Table S1. Medication Use. (DOCX 12 kb) [file 12979_2019_143_MOESM1_ESM.docx]

| **Supplementary Table 1: Medication Use** | | |
| --- | --- | --- |
| **Aged** | **Cohort 1** | **Cohort 2** |
| Statins | 0 | 2 |
| Metformin | 1 | 1 |
| Reflux | 1 | 2 |
| Thyroid | 1 | 1 |
| Allergy | 0 | 2 |
| Vitamin | 3 | 4 |
| **Young** | **Cohort 1** | **Cohort 2** |
| Allergy | 2 | 2 |
| Birth Control | 1 | 2 |
| Seizure | 1 | 1 |
| Vitamin | 2 | 5 |

Numbers denote number of subjects reporting use
of medications of the listed class.
